# THE THERAPEUTIC IMPACT OF PROBIOTICS ON NONALCOHOLIC FATTY LIVER DISEASE IN PEDIATRICS: A SYSTEMATIC REVIEW

**DOI:** 10.1590/1984-0462/2021/39/2019226

**Published:** 2020-08-26

**Authors:** Felipe Galvão Batista Chaves, Glauco Ferreira de Oliveira, João Paulo Ribeiro, João Victor Serafim, Luiz Felipe Medeiros Cordeiro, Matheus Alves Alvares, Marcelo Trindade Cecchi, Murilo Cordeiro Vasquez, Thaísy Bianka Dorta de Souza, Vera Esteves Vagnozzi Rullo

**Affiliations:** a Centro Universitário Lusíada, Santos, SP, Brazil.

**Keywords:** Non-alcoholic fatty liver disease, Pediatrics, Pediatric obesity, Probiotics, Liver steatosis, Doença hepática gordurosa não alcoólica, Pediatria, Obesidade infantil, Probióticos, Esteatose hepática

## Abstract

**Objective:**

Evaluate the effects of probiotics use, compared with placebo, in pediatric patients with non-alcoholic fatty liver disease (NAFLD), using laboratorial and ultrasonographic parameters as outcomes.

**Methods:**

A systematic review of the literature was performed through MEDLINE and Lilacs databases. The articles selected were randomized controlled clinical trials published until November 2018, without any language restriction, dealing with pediatric patients with NAFLD. Patients were divided into 2 groups. One group received probiotic therapy and the other group, only received placebo. The primary outcome evaluated was the difference between the serum levels of alanine aminotransferase (ALT) before and after receiving probiotics or placebo. The secondary outcomes evaluated were the serum aspartate aminotransferase levels, body mass index, serum triglycerides, waist circumference and level of liver steatosis on the ultrasonography.

**Results:**

A total of 46 articles were recovered, and 3 articles were included in the qualitative analysis, totaling 128 patients. Two trials revealed a significant decrease of alanine aminotransferase levels after treatment with probiotics (*Lactobacillus rhamnosus* for 8 weeks; *Bifidobacterium+Lactobacillus* for 12 weeks), when compared to the placebo. The other variables did not show a statistically significant difference between both groups.

**Conclusions:**

Probiotic therapy has contributed to the reduction of ALT serum levels in pediatric patients with nonalcoholic fatty liver disease, which is in line with results found by other authors in scientific literature. Regarding the secondary outcomes, the use of probiotics did not show benefits or damages compared to placebo.

## RESUMO

**Objetivo:**

Avaliar os efeitos do uso de probióticos em comparação com placebo, em pacientes pediátricos portadores de doença hepática gordurosa não alcoólica (DHGNA), utilizando parâmetros laboratoriais e ultrassonográficos como desfecho.

**Métodos:**

Revisão sistemática da literatura por meio das bases de dados Sistema Online de Busca e Análise de Literatura Médica (MEDLINE) e Literatura Latino-Americana e do Caribe em Ciências da Saúde (Lilacs). Foram selecionados ensaios clínicos controlados randomizados publicados até novembro de 2018, sem restrição de língua, com pacientes pediátricos portadores de DHGNA, divididos em dois grupos. Um grupo foi submetido à terapia probiótica e outro grupo recebeu somente placebo. O desfecho primário avaliado foi a comparação dos níveis de alanina aminotransferase (ALT) ao início e no fim do seguimento entre os grupos probiótico e placebo. Os desfechos secundários avaliados foram os níveis de aspartato aminotransferase sérico, índice de massa corpórea, triglicerídeos totais séricos, circunferência abdominal e grau de esteatose hepática à ultrassonografia abdominal.

**Resultados:**

Foram recuperados 46 artigos, sendo três incluídos na análise qualitativa, totalizando 128 pacientes. Dois estudos demonstraram redução significativa dos níveis de ALT com o uso de probiótico (*Lactobacillus rhamnosus*, por oito semanas; *Bifidobacterium+Lactobacillus*, por 12 semanas), em comparação ao placebo. As demais variáveis avaliadas não evidenciaram diferença estatisticamente significante ente os dois grupos.

**Conclusões:**

O uso de probióticos representou redução nos níveis séricos de ALT na esteatose hepática na infância, indo ao encontro dos resultados obtidos por outros autores da literatura científica vigente. No que se refere às variáveis de desfecho secundário, não foi demonstrado benefício ou dano do tratamento de probióticos em relação ao placebo.

## INTRODUCTION

The latest data from the World Health Organization (WHO) indicate that childhood obesity is growing globally, and is a public health problem that already affects one fifth of the world’s children.^1^ ^,^ ^2^ Worldwide, there will be an increase in the number of obese children, totaling around 70 million in 2025, according to the WHO.^2^ The WHO Commission on Ending Childhood Obesity (ECHO) estimates that the number of obese or overweight preschoolers went from 32 million in 1990 to 41 million in 2016, for a total of 340 million children and adolescents in these conditions that year.^3^ ^,^ ^4^ Most live in underdeveloped or developing countries, whose rates of overweight and obesity have increased by 30% compared to developed countries.^3^ ^,^ ^5^ In the richest countries, despite the reduction in the growth rate of childhood obesity, prevalence has not decreased.^6^ ^,^ ^7^

Without an intervention, obese children tend to become obese adults, just as overweight young people can become obese adults.^3^ ^,^ ^8^ The incidence of type 2 diabetes mellitus, coronary heart disease, high blood pressure, some types of cancer and osteoarticular problems is also more likely.^8^ ^,^ ^9^ In addition, certain manifestations may have an effect in the short term, such as dyslipidemia, insulin resistance and non-alcoholic fatty liver disease (NAFLD).^9^

Considering this, NAFLD has an average prevalence of 7.6% (95% confidence interval [95%CI] 5.5-10.3%) in the general pediatric population.^10^ When specifically analyzing obese children, this number is on average 34.2% (95%CI 27.8-41.2%), and additionally, a higher prevalence in boys than in girls (ratio 2:1) has been found.^10^ It was also observed that prevalence is higher as body mass index increases (BMI z score).^10^

This condition can be defined as excessive formation of adipose tissue in the hepatic parenchyma, leading to the process of hepatic steatosis in the absence of secondary causes such as alcoholism, hepatitis C, parenteral nutrition, errors of metabolism, apnea obstructive sleep, among others.^11^

New evidence contributes to the understanding of its pathophysiology and demonstrates the role of the intestinal microbiota in the production of these reactive species. Furthermore, it shows pro-inflammatory substances, the expression of nuclear factors and cytokines that contribute to the development of NAFLD and its progression to steatohepatitis and hepatic fibrosis, and that some of these substances can even be detected in the early stages of NAFLD in children.^12^ ^,^ ^13^

In addition to traditional therapeutic options, experimental studies indicate that the use of prebiotics, probiotics and symbiotics in the modulation of the intestinal microbiota proved beneficial in the treatment of obesity and NAFLD.^14^ ^,^ ^15^ ^,^ ^16^ ^,^ ^17^ ^,^ ^18^ However, some scientific societies argue that the number of evidence strains on probiotic treatments is poor and further studies are needed in the population to understand their risks and benefits in a greater number of individuals.^19^ Similarly, there is no indication that they have been used in the latest evidence-based treatment algorithms from leading specialized societies around the world.^19^ ^,^ ^20^ ^,^ ^21^

Therefore, the present study aimed to provide an updated analysis of the use of probiotics in HDNGA in childhood, because they do not yet have their therapeutic impact fully elucidated with regard to the pediatric population.

## METHOD

This systematic review of randomized controlled trials evaluated the effect of probiotic therapy on NAFLD in childhood. For the selection of trials, a systematic search was carried out in primary databases, including Online Search and Analysis System of Medical Literature (MEDLINE), via PubMed, and Latin American and Caribbean Literature in Health Sciences (Lilacs), via the Virtual Health Library (VHL).^22^ ^,^ ^23^ For MEDLINE, the following search strategy was used: (Prebiotics OR probiotics OR lactobacillus OR bifidobacterium) AND “liver diseases” [MeSH Terms] AND (adolescent OR child OR “child, preschool” OR infant OR “infant, newborn”). For Lilacs, the following search strategy was used: (probiotics AND liver).

Experimental randomized controlled trial studies were included, comparing the use of probiotics with the non-use of these products for NAFLD in pediatric age groups. The following articles were excluded from the systematic review:

- Observational Studies.
- Studies with a non-pediatric age group.
- Literature reviews.
- Studies performed on animals.
- Duplicates

After the systematic search in the literature through the MEDLINE and Lilacs databases, articles that met the inclusion and exclusion criteria of the study were selected. Only complete publications were included, and there was no language restriction. A total of 47 papers were found: 31 through MEDLINE and 16 via Lilacs. The search was carried out until October 2018.

**Figure 1:**
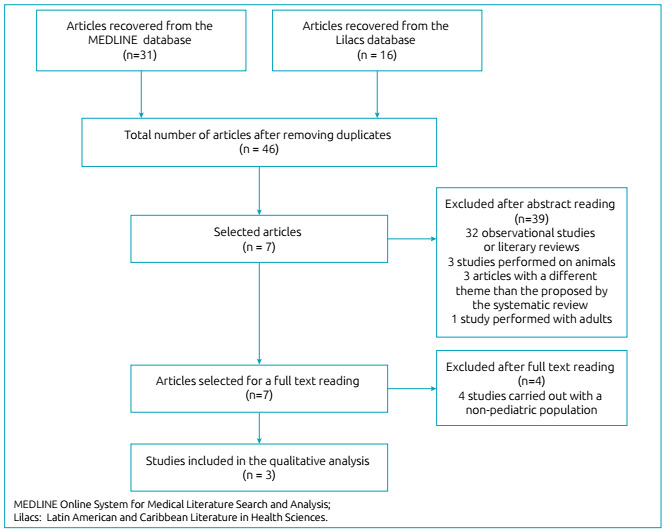
Flow diagram of the selection of studies included in the analysis.

After reading the abstracts of all articles, 40 studies were excluded from the qualitative analysis, 32 of which were observational studies/literature reviews; three were developed with animals; three did not evaluate patients with nonalcoholic liver disease; one analyzed only adult patients; and a duplicate article, which was found both in the MEDLINE database and in the Lilacs database. Thus, seven randomized clinical trials were selected for full text analysis. During this stage, four studies were excluded from the selection, because they presented a non-pediatric study population. The remaining three studies were included in this systematic review. The process of selecting articles can be seen in Figure 1.

In order to evaluate the prognostic impact on NAFLD, the three clinical trials included in this analysis used objective criteria for the follow-up of children with hepatopathy. These criteria were based on laboratory tests of liver function, ultrasound findings, body mass index (BMI) and percentage of fat in liver tissue.

In this review, the primary endpoint was the serum value of alanine aminotransferase (ALT), which was observed in the three articles. Secondary outcomes were serum values of aspartate aminotransferase (AST) and triglycerides and z score for BMI.

## RESULTS

The three selected articles were randomized clinical trials that adopted patients taking probiotics for an intervention group and patients receiving only placebo for the control group. Patients were equally allocated between the probiotic group and the placebo group. The object of study was school-age children and adolescents with NAFLD, totaling 128 subjects in the three trials evaluated. The individual characteristics of the studies are expressed in Table 1. The quality criteria of the selected randomized clinical trials were adapted from the recommendations proposed by the Scottish Intercollegiate Guidelines Network (SIGN) and can be observed in Table 2.^24^

**Table 1:** Characteristics of the selected studies.

Article	Vajro et al.^25^	Alisi et al.^26^	Famouri et al.^27^
Publication year	2011	2014	2017
Probiotic	*Lactobacillus rhamnosus*	VSL#3	*Bifidobacterium* + *Lactobacillus*
Probiotic group	10	22	32
Placebo group	10	22	32
Total sample	20	44	64
Follow-up time	8 weeks	4 months	12 weeks

**Table 2:** Quality criteria of clinical trials included in the study as adapted from the recommendations of the Scottish Intercollegiate Guidelines Network (SIGN).

Article	Vajro et al.^25^	Alisi et al.^26^	Famouri et al.^27^
Focused and appropriate clinical issue	Yes	Yes	Yes
Randomization	Yes	Yes	Yes
Allocation prohibited	Yes	Yes	Yes
Double blind study	Yes	Yes	Yes
Initial homogeneity between the groups	Not reported	Yes	Yes
Probiotic as the only difference between the groups	Yes	Yes	Yes
Outcomes measured using a reliable method	Yes	Yes	Yes
Losses	0%	8.4%	Not reported
Analysis by intention to treat	Yes	Yes	Yes
Multicenter study	No	No	No
Bias minimization	Yes	Yes	Yes

Twenty patients participated in the study conducted by Vajro et al.^25^ All of them were obese children (age 10.7±2.1 years old) (BMI higher than the 95th percentile for age and sex) and patients with NAFLD. Inclusion criteria in the study were the persistence of serum ALT greater than 40 IU/L for at least three months and the presence of hepatic steatosis on an ultrasound. Individuals with other etiologies for liver disease were excluded. Patients were allocated into two groups of 10. The first group underwent oral therapy with probiotic *Lactobacillus rhamnosus*, while the second received placebo. Follow-up occurred after eight weeks, and the primary outcome evaluated was the ALT serum level. Other variables observed by the study were BMI, concentration of tumor necrosis factors alpha (TNF-alpha) and test results using IgA serum antibodies for peptidoglycan-polysaccharides (PG-PS IgA) complexes. To evaluate the liver on the ultrasound, the authors analyzed the echogenicity of the parenchyma, how it penetrates in deep hepatic tissues, and visualizing the vascular structures of the organ. Liver textures were quantitatively compared to renal echogenicity, allowing for an evaluation of the hepatorenal ultrasound relationship as a parameter for assessing hepatic fatty impairment. In the evaluation of TNF-alpha and PG-PS IgA levels and the hepatorenal ultrasound relationship, the values obtained in the study were compared to those of infant populations without liver diseases.

Alisi et al.^26^ conducted an experimental study with 44 obese children (percentile for BMI>85 for age and sex) with nonalcoholic fatty liver disease. The mean age of the patients was 10.5 years old. Individuals with borderline serum ALT values below 40 IU/L with no signs of any other cause of liver disease were selected. Participants were randomized into groups of 22, receiving the VSL#3 probiotic or placebo for four months. Children up to nine years of age in the probiotic group received one sachet per day, while those aged ten years or more ingested two sachets. The main outcome evaluated was the severity of the liver disease from the ultrasound, defined in a graduation from zero to three, where grade zero indicates normal liver and other degrees in mild, moderate and severe liver disease, depending on the intensity of increased echogenicity of the hepatic parenchyma and how the diaphragmatic edge and portal vein look. After the four-month follow-up, the results were computed and a mathematical regression model demonstrated the evolution of the ultrasound results by means of probabilities. Other outcomes considered by this trial included ALT levels and glucagon-like peptide (GLP-1) levels and BMI changes. The insulin resistance level of both groups was also evaluated using the homeostasis evaluation model, known as homeostasis model assessment - insulin resistance (HOMA-IR), obtained by the equation: fasting insulin×fasting glucose/405 (in mg/dL).

In turn, Famouri et al.^27^ evaluated a population of 64 individuals between ten and 18 years old. Only those with ultrasound evidence of nonalcoholic fatty liver disease and with a BMI equal to or greater than the 85^th^ percentile for age and gender were included. Patients with liver diseases due to other etiologies were excluded. By random allocation, 32 patients underwent probiotic therapy and another 32 only received placebo during the study period. Probiotic therapy was based on a capsule containing bacteria of the genera *Lactobacillus* and *Bifidobacterium*. Follow-up was 12 weeks long. The variables evaluated by the study were ALT serum, AST serum, lipid profile (given by the evaluation of low-density lipoproteins, high density lipoproteins and triglycerides) and waist circumference. The degree of fatty liver disease was also evaluated using the same classification method used by Alisi et al., except for the use of a mathematical model and probabilities.

The elevation of hepatic transaminases such as ALT is a frequent laboratory result of liver disease. This laboratory marker can be adopted as an indirect control of the progression of NAFLD. Thus, serum ALT values were evaluated before and after probiotic therapy compared to placebo. The results are presented in Table 3 and refer to the mean of the values found among the participants of each study and their respective standard deviation.

**Table 3:** Serum concentration of alanine aminotransferase (IU/L) before and after follow-up.

Article	Probiotic		Placebo		p-value*
	Initial	Final	Initial	Final	
Vajro et al.^25^	70.3 (34.8)	40.1 (22.4)	63.6 (18.5)	61.6 (31.8)	0.03
Alisi et al.^26^	34.0 (1.0)	33.0 (1.0)	42.0 (1.0)	50.0 (5.0)	0.17
Famouri et al.^27^	32.8 (19.6)	23.1 (9.6)	28.9 (13.7)	26.2 (12.9)	<0.05

*Comparisons between the groups at the end of the tests.

The mean ALT concentrations between the control group and the probiotic group were similar in the baseline measurement conducted by Vajro et al., showing initial mean serum concentration of 63.6 IU/L in the control group and 61.6 IU/L in the probiotic group. Famouri et al. found mean baseline ALT levels of 28.9 IU/L in the placebo group and 32.8 IU/L in the probiotic-treated group. In turn, Alisi et al. obtained initial measurements with greater differences. They showed mean values of 42 IU/L in the control group against 34 IU/L in the group treated with VSL#3. As for the results achieved after follow-up of the groups, Vajro et al. and Famouri et al. demonstrated a decrease in the concentration of more expressive ALT in the probiotic group in relation to patients receiving placebo and with statistically relevant results (p=0.03 and p<0.05, respectively).^25^ ^,^ ^27^ On the other hand, Alisi et al. revealed that the reduction was not significant in the probiotic group and there was a direct increase in ALT levels in the placebo group. None of the results obtained statistical significance (p=0.17).^26^

In addition to ALT, evaluated by the three clinical trials, other markers were used in the evaluation of nonalcoholic liver disease in pediatrics, such as ultrasound findings, AST serum concentrations, mean triglyceride concentration, and mean BMI (Z-score or z-BMI). All variables were evaluated at the beginning and end of the follow-up of the studies. Table 4 displays the results obtained for the secondary markers in their respective studies.

**Table 4:** Secondary outcomes assessed by the selected studies.

Marker	Article	Probiotic		Placebo		p-value^†^
		Initial	Final	Initial	Final	
USG	Vajro et al.^25^*	1.31 (0.26)	1.30 (0.15)	1.17 (0.12)	1.22 (0.12)	>0.05
	Famouri et al.^27^**	0% Normal	53.1% Normal	0% Normal	16.5% Normal	<0.05
		62.5% Grade I	25.0% Grade I	56.2% Grade I	46.9% Grade I	
		37.5% Grade II	21.9% Grade II	43.8% Grade II	37.5% Grade II	
	Alisi et al.^26^***	55% moderate 45% severe	21% normal 70% mild 9% moderate 0% severe	64% moderate 36% severe	0% normal 7% mild 76% moderate 17% severe	<0.001
AST (IU/L)	Famouri et al.^27^	32.2 (15.7)	24.3 (7.7)	30.2 (12.9)	26.6 (11.8)	<0.05
TG (mg/dL)	Alisi et al.^26^	99.0 (4.0)	110.0 (9.0)	98.0 (3.0)	102.0 (10.0)	0.575
	Famouri et al.^27^	112.5 (50.5)	100.6 (44.8)	96.03 (20.6)	91.9 (19.4)	<0.001
BMI (Z score)	Vajro et al.^25^	2.29 (0.30)	2.21 (0.31)	2.12 (0.24)	2.00 (0.26)	>0.05
	Alisi et al.^26^	1.94 (0.01)	1.58 (0.04)	1.68 (0.01)	1.68 (0.01)	<0.001
WC (cm)	Famouri et al.^27^	82.2 (14.7)	80.3 (15.1)	81.4 (6.8)	80 (7.2)	>0.05

USG: ultrasound; AST: aspartate aminotransferase; TG: triglycerides; BMI: body mass index; WC: waist circumference; † value between groups at the end of the trials; *Vajro et al.^25^ used the hepatorrenal ultrasound relationship as an evaluation method; **Famouri et al.^27^ classified hepatic steatosis from an ultrasound in degrees of impairment; ***Alisi et al.^26^ associated ultrasound findings with a mathematical model to obtain the chances of categorizing patients after four months of study.

As for the ultrasound study, Vajro et al. did not find significant changes in ultrasound findings between the control and probiotic groups at eight weeks of follow-up (p>0.05).^25^ Alisi et al. reported beneficial changes at the end of four months of probiotic supplementation. At the end of the study, a mathematical simulation was performed, which demonstrated that the chances of patients treated with probiotics not presenting fatty liver were 21%, 70% for mild satosis, 9% for moderate steatosis and 0% for the severe form. In the placebo group, comparatively, these odds were 0, 7, 76 and 17%, respectively.^26^

Famouri et al., in turn, reported beneficial changes with regard to the degree of steatosis of patients who received probiotics after 12 weeks. There was an increase in the percentage of patients without changes in the ultrasound, as well as a reduction in the number of patients classified as grade I and grade II. The changes presented statistically significant values (p<0.05).^27^

The work of Famouri et al. was also the only one to follow the AST serum concentration of patients. As observed in Table 4, this variable was reduced in both study groups. This decrease, however, was more pronounced in the group undergoing probiotic therapy, with a drop of 7.9 IU/L after 12 weeks of follow-up, against only 3.6 IU/L in the placebo group (p<0.05). ^27^

Two articles followed the concentration of triglycerides in both groups at the beginning and end of the follow-up. Comparatively, it was observed that the studies show contradicting and little relevant results regarding therapeutic impact. Alisi et al. showed an increase in TG levels in both groups, with no statistical significance (p=0.575). However, in the study by Famouri et al., there was a slight reduction in the two groups studied (p<0.001).^26^ ^,^ ^27^

Regarding BMI, in Vajro et al., no significant variation was seen in the studied population regardless of the treatment adopted (p>0.05). Alisi et al., in turn, observed a moderate reduction in BMI after probiotic treatment, while there was no variation in body mass in the placebo group (p<0.001). The standard deviation (Z score) of BMI for age and gender was used as a reference. Finally, Famouri et al. used waist circumference (cm) as an alternative parameter to BMI to control obesity in the population studied. There was no benefit of the intervention in reducing this variable in the probiotic group compared to the placebo group (p<0.05). However, comparing the baseline and final values within the probiotic group alone, a reduction was obtained with significance (p=0.001), contrary to what was observed in the control group (p=0.06).^27^

## DISCUSSION

The reduction in ALT levels demonstrated mainly by Vajro et al. and Alisi et al. is in line with what is reported in the world literature. Lavekar et al. developed a meta-analysis with seven experimental studies in the pediatric population with NAFLD and found a significant drop in ALT levels in all of the studies (ALT=-20.97 IU/L; CI95% -36.14--5.81; p<0.0001).^28^ Another meta-analysis, conducted by Yan Ma et al. with four randomized clinical trials involving 134 patients, also confirmed a reduction in ALT with statistical significance (ALT=- 23.71 IU/L; CI95% -33.46--13.95; p<0.00001).^29^

However, other markers were also used heterogeneously among the studies present in this review, which hinders their joint analysis. Only Famouri et al. used AST as an evaluable parameter and demonstrated a greater drop in their levels in the probiotic group compared to the placebo group, although there was a similar drop between the groups. The meta-analyses conducted by Lavekar et al. and Yan Ma et al. also showed a reduction in the levels of this enzyme (AST=-19.24 IU/L; CI95% -28.75--9.7; p<0.0001; and AST=-19.77 IU/L; CI95% -32.55--7.00; p=0.002).^28^ ^,^ ^29^ Therefore, there is evidence of a reduction in AST that is higher than placebo, although more evidence is needed.

BMI was assessed by Vajro et al. and Alisi et al. While the study by Alisi et al. indicated greater negative variation with the use of probiotic, Vajro et al. did not observe a significant difference between the intervention group and the placebo group (range -3.5% versus -5.7%, respectively). In the world literature, however, there is still controversy regarding the effects of probiotics on BMI, with some authors confirming alterations and others reporting no changes, both in the adult and pediatric population.^30^ ^,^ ^31^ ^,^ ^32^ There are even conflicts between meta-analyses.^28^ ^,^ ^29^

In addition to the heterogeneity of markers among the studies selected in this review, the small number of studies obtained, which is associated with the small samples in each, impairs the analysis when comparing with other studies. In the BMI assessment, for example, the short evaluation period associated with the small sample is one of the factors that hinder the precise analysis of this variable.

The fact that the studies were not carried out in multiple centers also limits their application as an evaluating tool of different populations, and shows there is a need for broader research on the applicability of probiotic therapy in children and adolescents with NAFLD.

In the future, investigations with longer follow-up time and more rigorous evaluation of the individual performance of participants regarding changes in life habits related to obesity and being overweight will be essential. This may generate bias in the results of the studies. An alternative to reduce biases in this sense would be to categorize the participants according to age group, taking into account the degree of understanding about the disease, causal factors and complications.

In conclusion, the drop in ALT serum levels found in the selected clinical trials corroborates the results obtained by other authors in systematic reviews and meta-analyses. In relation to serum levels of AST, triglycerides and BMI, no benefit or damage of probiotic treatment has been demonstrated with regard to placebo, however there is heterogeneity among probiotics used in the selected studies, as well as in the parameters evaluated, making it difficult to have a more comprehensive systematic analysis. In addition, there are few randomized clinical trials that are suitable for comparative effect regarding the therapeutic impact, when using the selection methods proposed by the present study. Thus, more evidence is needed to more accurately elucidate the advantages of probiotic therapy in the management of GHNAD in childhood.
